# Cervico-thoracic Interspinous Bursitis Associated with Bilateral Upper-extremity Numbness: A Case Report

**DOI:** 10.7759/cureus.1897

**Published:** 2017-11-30

**Authors:** Ronen Blecher, Emre Yilmaz, Tamir Tawfik, Amir Abdul-Jabbar, Thomas O'Lynnger, Marc Moisi, Robert Hart, David Hanscom, Rod J Oskouian, R. Shane Tubbs, Jens Chapman

**Affiliations:** 1 Swedish Medical Center, Swedish Neuroscience Institute; 2 Neurosurgery, Swedish Neuroscience Institute; 3 Neurosurgery, Seattle Science Foundation; 4 Orthopedics Spine Surgery, Swedish Neuroscience Institute

**Keywords:** inter-spinous, bursitis, spine

## Abstract

The authors describe a 48-year-old woman suffering from bilateral upper-extremity numbness and axial radiating pain. Magnetic resonance imaging revealed soft-tissue edema and enhancement surrounding the dorsal tip of the C7 spinous process. Excisional biopsy of the lesion revealed a mildly inflamed bursa, with no evidence of an active infection. Removal of the inflamed bursa resulted in complete resolution of the upper-extremity numbness and improvement in her neck pain. Although similar cases have been reported to be associated with rheumatologic conditions, most notably polymyalgia rheumatica (PMR), the current report underlines the presentation of radicular-like complaints associated with interspinous bursitis in the absence of other conditions affecting the musculoskeleton.

## Introduction

The spinal column harbors a variable number of bursae, usually located between each of the spinous processes of the cervical and lumbar segments [1]. Inflammation of these structures may result in substantial and occasionally overlooked causes of neck or back pain. In the cervical spine, interspinous bursitis has been commonly associated with rheumatic conditions such as polymyalgia rheumatica (PMR) [2-7], rheumatoid arthritis, and crystalopathies [1]. In the lumbar spine, Baastrup’s disease, also known as “kissing-spine” syndrome, is a condition in which degenerative segmental changes may result in repetitive strains between adjacent spinous processes, local inflammation of the interspinous bursae, and bony erosions [8]. A multitude of evidence has thus far described the incidence, radiographic findings, and natural history of cervical interspinous bursitis that accompany PMR [2-7]. Here we present a case in which interspinous bursitis involving the cervical spine resulted in radicular-like symptoms in the absence of an identifiable rheumatologic condition.

## Case presentation

History and examination

**Figure 1:**
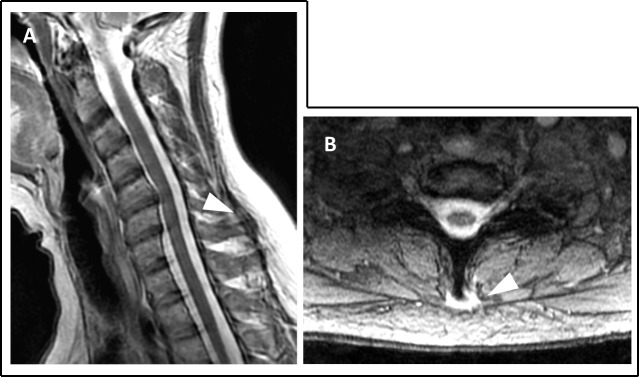
Magnetic resonance imaging (MRI) T2-sequence images of the cervical spine, sagittal, and axial views (A, B) MRI of the cervical spine on both the sagittal (A) and axial (B) views failed to reveal any gross abnormalities involving the soft tissues or neural elements. Retrospectively, no detectable abnormality was evident around the C7 spinous process in the sagittal plane (A; white arrowhead). Although detectable in the axial T2 images, the high signal around the spinous process of C7 (B; white arrowhead) was not striking and was therefore probably overlooked due to being non-specific.

**Figure 2:**
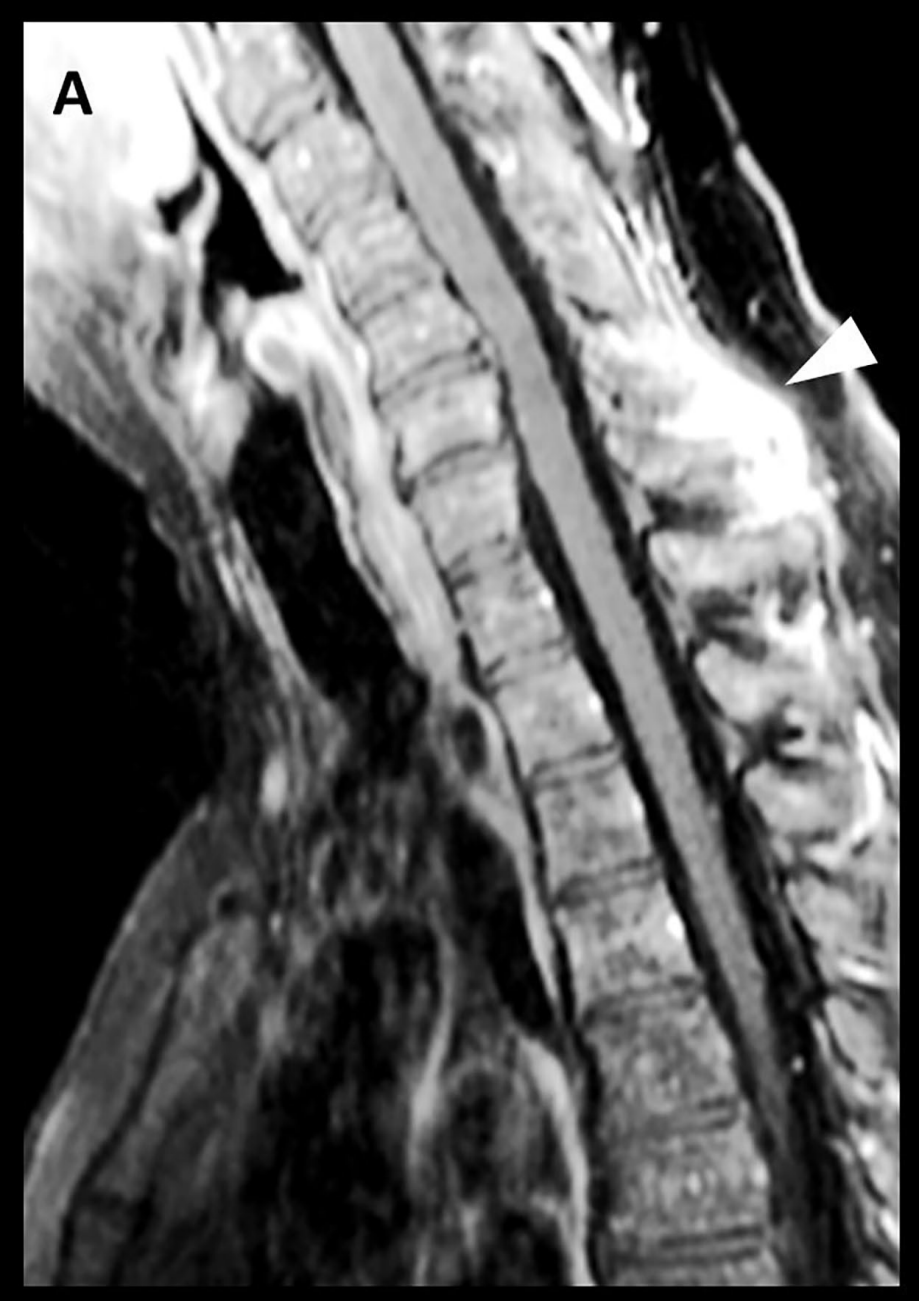
Magnetic resonance imaging (MRI) with contrast of the cervical spine, sagittal view (A) Whole-spine MRI, this time with contrast, clearly revealed an increased contrast enhancement around the C7 spinous process, suggestive of a possible soft-tissue inflammatory process or an abscess formation (white arrowhead).

A 48-year-old woman presented to the emergency department (ED) with complaints of neck pain, right-sided facial and hand pain, hand numbness, intermittent fever, and a bulge at the posterior aspect of her neck. The patient’s past history was found to be positive for removal of a pituitary macro-adenoma, chronic headaches, neck and back pain as well as occasional upper-extremity numbness. Physical examination revealed a reduced cervical range of motion and spinous process tenderness. No neurologic deficits were found. Workup to rule out infection including white blood cell count, C-reactive protein, and repeated lumbar punctures was negative. Initial diagnostic imaging which consisted of cervical spine magnetic resonance imaging (MRI) without contrast demonstrated mild-to-moderate degenerative changes of the cervical spine without findings suggestive of neural or soft tissue infection (Figure 1). Following two weeks of failed conservative treatment, and due to worsening complaints of pain and numbness of her posterior neck and hands, imaging evaluation was expanded. Whole-spine MRI, this time with contrast (Figure 2), revealed soft-tissue edema and enhancement surrounding the tip of the C7 spinous process.

Surgical treatment and post-operative course

Following evaluation, the patient underwent excisional biopsy of the C7 peri-spinous process soft tissue. Exploration revealed mildly inflamed bursa with no evidence of infection or involvement of other soft or osseous elements. Following excision, the patient reported significant improvement in neck pain and hand numbness. The bursa specimens sent for cultures, including acid-fast bacilli and fungi, yielded no growth.

## Discussion

In an anatomic study performed in 27 cadavers by Bywaters et al. [1], the incidence of cervical interspinous bursae approached 50% and was found to usually involve the C6-C7 processes. Although relevant data is lacking, we believe that it is only reasonable to assume that this rate is likely higher in populations at risk for the presence of cervical bursitis, such as patients complaining of neck pain. In addition, several studies also established a clear association between inter-spinous bursitis of the cervical spine and other rheumatologic conditions, most notably polymyalgia rheumatica, suggesting a diagnostic role for cervical spine bursitis [5-7]. On a broader perspective, awareness of performing unnecessary spine surgery has grown considerably during recent years among patients, surgeons, and hospitals [9]. In that context, identifying other pain generators in the neck may contribute both to reducing the rate of performing unnecessary spinal procedures but also in providing patients with better care.

## Conclusions

In conclusion, this case, albeit consisting of merely a “minor” nuisance, may provide important lessons in a broader context. First, we suggest that interspinous bursitis should be considered as one of the more common pain generators during evaluation of patients complaining of either simple or “radicular-like” neck pain. Simple palpation of the spinous processes during physical exams, and looking for signs of increased contrast uptake may assist in the diagnosis. Second, the high association previously reported between interspinous bursitis and multi-system rheumatologic pathologies, such as PMR, may serve to both prompt further rheumatologic evaluation as well as assist in avoiding unnecessary surgical interventions for other non-surgical pathologies.
